# Bacterial Endophytes of Spring Wheat Grains and the Potential to Acquire Fe, Cu, and Zn under Their Low Soil Bioavailability

**DOI:** 10.3390/biology10050409

**Published:** 2021-05-05

**Authors:** Orysia Makar, Agnieszka Kuźniar, Ostap Patsula, Yana Kavulych, Volodymyr Kozlovskyy, Agnieszka Wolińska, Ewa Skórzyńska-Polit, Olena Vatamaniuk, Olga Terek, Nataliya Romanyuk

**Affiliations:** 1 Department of Plant Physiology and Ecology, Ivan Franko National University of Lviv, 4 Hrushevsky Street, 79005 Lviv, Ukraine; orysia.makar@lnu.edu.ua (O.M.); ostap.patsula@lnu.edu.ua (O.P.); yana.kavulych@lnu.edu.ua (Y.K.); olha.terek@lnu.edu.ua (O.T.); 2 Department of Biology and Biotechnology of Microorganisms, The John Paul II Catholic University of Lublin, 1I Konstantynów Street, 20-708 Lublin, Poland; agnieszka.wolinska@kul.pl; 3 Institute of Ecology of the Carpathians, NAS of Ukraine, 4 Kozelnytska Street, 79000 Lviv, Ukraine; vkozlovskyy@gmail.com; 4 Department of Plant Physiology and Biotechnology, The John Paul II Catholic University of Lublin, 1I Konstantynów Street, 20-708 Lublin, Poland; eskorzynska@kul.pl; 5 Soil and Crop Sciences Section, School of Integrative Plant Science, Cornell University, 608 Bradfield Hall, Ithaca, NY 14853, USA; okv2@cornell.edu

**Keywords:** spring wheat, *T. aestivum* L., emmer wheat, yield, endophytic bacteria, iron, copper, zinc

## Abstract

**Simple Summary:**

Unmasking the overall endophytic bacteria communities from wheat grains may help to identify and describe the microbial colonization of bread and emmer varieties, their link to the bioactive compounds produced, and their possible role in mineral nutrition. The possibility of using microorganisms to improve the microelemental composition of grain is an important food security concern, as approximately one-third of the human population experiences latent starvation caused by Fe (anemia), Zn, or Cu deficiency. Four wheat varieties from *T. aestivum* L. and *T. turgidum* subsp. *dicoccum* were grown in field conditions with low bioavailability of microelements in the soil. Varietal differences in the yield, yield characteristics, and the grain micronutrient concentrations were compared with the endophytic bacteria isolated from the grains. Twelve different bacterial isolates were obtained that represented the genera *Staphylococcus*, *Pantoea*, *Sphingobium*, *Bacillus*, *Kosakonia*, and *Micrococcu*s. All studied strains were able to synthesize indole-related compounds (IRCs) with phytohormonal activity. IRCs produced by the bacterial genera *Pantoea* spp. and *Bacillus* spp. isolated from high-yielding Oksamyt myronivs’kyi and Holikovs’ka grains may be considered as one of the determinants of the yield of wheat and its nutritional characteristics.

**Abstract:**

Wheat grains are usually low in essential micronutrients. In resolving the problem of grain micronutritional quality, microbe-based technologies, including bacterial endophytes, seem to be promising. Thus, we aimed to (1) isolate and identify grain endophytic bacteria from selected spring wheat varieties (bread Oksamyt myronivs’kyi, Struna myronivs’ka, Dubravka, and emmer Holikovs’ka), which were all grown in field conditions with low bioavailability of microelements, and (2) evaluate the relationship between endophytes’ abilities to synthesize auxins and the concentration of Fe, Zn, and Cu in grains. The calculated biological accumulation factor (BAF) allowed for comparing the varietal ability to uptake and transport micronutrients to the grains. For the first time, bacterial endophytes were isolated from grains of emmer wheat *T. turgidum* subsp. *dicoccum*. Generally, the 12 different isolates identified in the four varieties belonged to the genera *Staphylococcus*, *Pantoea*, *Sphingobium*, *Bacillus*, *Kosakonia*, and *Micrococcu*s (NCBI accession numbers: MT302194—MT302204, MT312840). All the studied strains were able to synthesize the indole-related compounds (IRCs; max: 16.57 µg∙mL^−1^) detected using the Salkowski reagent. The IRCs produced by the bacterial genera *Pantoea* spp. and *Bacillus* spp. isolated from high-yielding Oksamyt myronivs’kyi and Holikovs’ka grains may be considered as one of the determinants of the yield of wheat and its nutritional characteristics.

## 1. Introduction

The production of hexaploid bread wheat (*Triticum aestivum* L.) around the world has reached around 750 million metric tons annually and thus wheat remains a key crop for the human food supply [1]. However, the grains of most cereal crops, including wheat, usually have low concentrations of essential micronutrients, such as zinc (Zn) and iron (Fe) [2,3,4,5,6,7,8]. Consequently, wheat grains are not a good source of these mentioned elements for combating micronutrient-deficiency-associated human health disorders that affect more than one-third of the world’s population [9,10,11]. In contrast, an ancient wheat variety, i.e., a tetraploid emmer wheat (*Triticum turgidum* subsp. *dicoccum*), is being increasingly recognized as a valuable food source for its high content of resistant starch, fiber, carotenoids, antioxidant compounds, and vitamins B2, B5, B6, and A [12,13,14,15,16]. Published reports have also indicated that wild and primitive wheats, such as *T. monococcum*, *T. dicoccon*, and *T. dicoccoides*, accumulate more Fe and Zn in grains than cultivated wheat and advanced lines [6,7,8]. However, our understanding of molecular and physiological mechanisms underlying this trait is still limited. It is also noteworthy that Fe, copper (Cu), and Zn deficiencies that occur in alkaline and organic soils, which occupy approximately 30% of arable land, significantly reduce wheat yields [17,18,19,20] and threaten food security [17,21,22].

Microbe-based technologies, including endophytes, i.e., plant-associated bacteria living in internal plant tissues, are gaining importance for improving soil properties and enhancing crop yield and the accumulation of nutrients in plant organs [23,24], especially in staple crops, such as wheat, maize, and rice [25,26]. While the past few decades were focused on the use of rhizosphere microorganisms to enhance the accumulation of micronutrients in grains [25,26,27], recently, the effect of endophytes on plant nutrition, growth, and vigor has attracted considerable attention [28,29,30]. The understanding of the microbial diversity and function in complex plant–soil environments has increased significantly as a consequence of the application of next-generation sequencing (NGS) methods [31,32]. It is well recognized that every plant species possesses its own endo-microbiome that works commensally or beneficially for the host [33,34,35]. Endophytic bacteria serve as rich sources of phytohormones and diverse secondary metabolites with a wide spectrum of biological activities [24,36,37,38,39,40,41,42,43,44]. An increasing number of scientific reports emphasize the important role of endophytes in priming plant immunity, removing contaminants, solubilizing phosphate, and contributing to nitrogen assimilation, thereby promoting plant growth and yield [45,46,47]. To date, most reports have been focused on the isolation of endophytic bacteria from different wheat organs and tissues [48,49,50]. A large diversity of endophytic bacteria belonging to different genera, including *Achromobacter*, *Acinetobacter*, *Arthrobacter*, *Bacillus*, *Chitinophaga*, *Enterobacter*, *Erwinia*, *Flavobacterium*, *Klebsiella*, *Leifsonia*, *Microbispora*, *Micrococcus*, *Micromonospora*, *Mycobacterium*, *Paenibacillus*, *Pantoea*, *Pseudomonas*, *Roseomonas*, *Staphylococcus*, *Streptomyces*, and *Xanthomonas* have been identified [51]. It has been evidenced that the microbial diversity decreases along the root–shoot axis, depending on the plant variety and the stage of plant growth [52]. Nowadays, many endophytic microorganisms are regarded as appropriate agents for enhancing Fe and Zn uptake and translocation. Promising results of enhanced Zn accumulation were obtained with the endophytes *Bacillus* spp., *Arthrobacter* spp. [37,53,54,55,56,57], and *Panthea* spp. [57]. The mechanisms promoting micronutrition that were specified by [58] include (1) organic acid secretion and proton extrusion; (2) indirect upregulation of Zn and Fe transporters; (3) secretion of phytohormone-like molecules, such as auxins (IAA), cytokinins, abscisic acid, brassinosteroids, ethylene, gibberellins, jasmonates, and strigolactones, as well as some specific amino acids [39,40,41,42,43,44,59]. As reported by White et al. [60], the inoculation of plant roots with endophytes produces wheat endophytic biota, microbial siderophores, and other mechanisms that sequester micronutrients efficiently; furthermore, metals adhere to microbial cell walls inside the plant root.

However, the knowledge of the role of particular genera of endophytic bacteria in the microelement acquisition by wheat in field conditions is insufficient [51,53,58,61]. Data regarding endophytes inhabiting spring wheat grains are equally scarce. It is worth emphasizing that grain endophytes are highly interesting due to their ability to be transmitted vertically between generations [62,63,64,65,66,67,68,69,70]. A better understanding and manipulation of endophytes are considered novel promising strategies to mitigate the adverse impacts of global climate change on agricultural production and to improve the nutritional quality of plant-based foods. In particular, beneficial wheat microbial strains can be considered as a sustainable tool not only for improving crop yields but also for increasing the micronutrient density in wheat grains, e.g., via biofortification [37,53,59,60,70,71,72,73,74].

Here, we aimed to isolate and identify grain endophytic bacteria from four selected spring wheat varieties, including emmer, which were all grown in field conditions with low bioavailability of microelements. We also aimed to evaluate the potential relationship between the abilities of the grain endophytes to synthesize auxins, grain yield characteristics, and the concentration of Fe, Zn, and Cu in grains. We hypothesized that some wheat grain bacterial endophytes have the ability to promote wheat growth and improve micronutrient content in grains. The presented data can help to provide novel strategies for biofortification applications and the improvement of food security.

## 2. Materials and Methods

### 2.1. Plant Material and Soil Characteristics

Grains of four wheat varieties—hexaploid bread wheat (*Triticum aestivum* L.), Oksamyt myronivs’kyi, Struna myronivs’ka, and Dubravka (V.M. Remeslo Myronivka Institute of Wheat of NAAS)—and one variety of domesticated tetraploid emmer wheat—*Triticum turgidum* L. subsp. *dicoccum* (Schrank ex Schübl.) Thell., i.e., Holikovs’ka (The Plant Production Institute V.Ya.Yuryev of NAAS)—were used in this study (State Register of Plant Varieties Suitable for Distribution in Ukraine, https://sops.gov.ua/reestr-sortiv-roslin, accessed on 6 March 2018).

Wheat plants were grown in four replications in a randomized complete block design during the 2017 vegetation period. The field experimental plots were located near Dmytriv village, Radekhiv district, Lviv region, Ukraine (50°13′26.6″ N, 24°36′50.5″ E). The plot surface area was 30 m^2^ (5 × 6 m) and the row spacing was 0.20 m. Grains were collected at the full ripening stage.

The soil type was Chernozem that formed on eluvium of carbonate rock, as described by Makar et al. [75]. The average soil pH was 7.15. The content of organic matter reached the level of 74.59 g∙kg^−1^. The concentrations of DTPA-extractable forms of micronutrients oscillated within the following ranges (ppm): Zn—1.22–4.44, Fe—8.23–18.29, and Cu—0.20–0.91 (Table 1).

### 2.2. Isolation of Bacterial Endophytes

The endophytic bacteria were isolated from *T. aestivum* L. and *T. turgidum* subsp. *dicoccum* grains following two sequential steps of surface sterilization. Specifically, the seeds were incubated for 20 min in a water: 4.5% sodium hypochlorite solution (1:1 ratio *v*/*v*), washed four times under running sterile water, and stratified at 4 °C for 24 h in sterile water in sterile beakers. Then, the grains were sterilized in the water:sodium hypochlorite solution (1:2 ratio *v*/*v*) for 15 min and rinsed four times with sterile water in sterile conditions.

The endophyte isolation was preceded by the analysis of the sterilization efficiency. The sterility of the wheat grains was controlled using indirect (culture on a general medium for bacteria, nutrient agar (BTL, Lodz, Poland)) and direct (polymerase chain reaction (PCR)) methods. The water from the last rinse of the grains was used as a template for the PCR as a control of the sterilization process. The description of the PCR conditions is provided in the section below.

The seeds were chopped using a sterile scalpel and half of the chopped samples were mixed with water. Both samples were placed in sterile Petri dishes with nutrient agar medium (BTL, Poland) that was supplemented with Nystatin (50 mg∙mL^−1^) and incubated (144 h, 30 °C, darkness). Subsequently, a single different colony that appeared on the solid medium was transferred onto a fresh nutrient agar medium (BTL, Lodz, Poland) and passed until pure strains were obtained, as confirmed using PCR and Sanger sequencing.

### 2.3. Identification of Endophytic Bacterial Strains

Bacterial DNA was extracted from the isolates using the method developed by Sambrook et al. [76] with modifications. Briefly, the cells were harvested via centrifugation at 17,500× *g* for 5 min (4 °C, Sigma 3–18 K, Sigma Laborzentrifugen GmbH, Osterode am Harz, Germany) and subjected to lysis in 5 mol·L^−1^ guanidine thiocyanate (Sigma-Aldrich, Saint Louis, MO, USA), 100 mmol/L EDTA (Sigma-Aldrich, Saint Louis, MO, USA), and 0.5% sarcosyl (Sigma-Aldrich, Saint Louis, MO, USA; pH 8.0). DNA was purified via extraction with ice-cold 7.5 mol·L^−1^ ammonium acetate (Sigma-Aldrich, Saint Louis, MO, USA) and subsequently using a chloroform:3-methyl-1-butanol (24:1, *v*/*v*) mixture (Sigma-Aldrich, Saint Louis, MO, USA). The two-phase mixture was centrifuged at 17,500× *g*. The upper layer was collected into a new tube. DNA was precipitated at −20 °C with 0.8 volumes of 2-propanol (Sigma-Aldrich, Saint Louis, MO, USA) for 1 h. The pellet was separated via centrifugation at 17,500× *g* for 30 min, rinsed five times with 70% (*v*/*v*) ethanol (Sigma-Aldrich, Saint Louis, MO, USA), dried under vacuum (RVC 2_18, Christ, Göttingen, Germany), and resuspended in 30 mL of ultrapure DNase-free water (free DNase, EURx, Gdańsk, Poland). The purity and concentration of the DNA were evaluated using a BioSpectrofotometer (Eppendorf, Hamburg, Germany).

PCR was performed in a reaction mixture containing 1× Phusion Flash High-Fidelity PCR Master Mix (Thermo Scientific, Waltham, MA, USA). The mixture also contained 1 µL of template DNA (in the range from 96.750 to 1278.190 µg∙mL^−1^ (Table A1), as well as sterile double-distilled water (free DNase, EURx, Gdańsk, Poland) in a total volume of 25 µL. In addition, universal eubacterial primers (each 1.0 µM): 27F and 1492R (Table A2, Genomed S.A., Warsaw, Poland) were applied. The PCR conditions were as follows: 98 °C for 10 s; 30 cycles of 95 °C for 5 s, 56 °C for 5 s, and 72 °C for 40 s (LABCYCLER, SensoQuest GmbH, Gdańsk, Germany). The PCR products were run on agarose gel (1%) and visualized with the use of SimplySafe™ (EURx, Gdańsk, Poland). Sterile double-distilled water (free DNase, EURx, Poland) was used as a negative control, while gDNA isolated from *E. coli* DH5α™ (Thermo Scientific, USA) was treated as a positive control. The PCR products were purified and sent to sequencing (Genomed S.A., Warsaw, Poland). The sequences were analyzed using the web version of the BLASTN algorithm (NCBI Bethesda, MD, USA) for the identification of the isolates from the seeds. The identified sequences were deposited in the GenBank (NCBI, http://www.ncbi.nlm.nih.gov/, accessed on 9 April 2020)) under the following accession numbers: MT302194—MT302204 and MT312840.

### 2.4. Production of the IRCs

The identified bacterial strains, namely, MT302194–MT302204 and MT312840 (*n* = 3), were incubated at 30 °C in darkness on a rotary shaker (125 revolutions per min (rpm)) in liquid nutrient broth (BTL, Lodz, Poland) supplemented with 5 mmol·L^−1^ of L-tryptophan. After 120 h, samples were pelleted via centrifugation at 10,000 rpm for 10 min and 2 mL aliquots of the supernatant were mixed with 4 mL of Salkowski’s reagent (50 mL 35% HClO_4_, 1 mL 0.5 M FeCl_3_·6H_2_O) [77]. The original formulation of the Salkowski reagent was used, as well as a standard time between the addition of the reagent and the reading of absorbance. The mixture prepared in this way was incubated at 30 °C for 30 min in darkness. The concentration of IRCs was measured colorimetrically at 530 nm (BioSpectrofotometer, Eppendorf, Germany) using a calibration curve for the indole acetic acid (IAA) standard ranging up to 100 µg·mL^−1^ (Sigma-Aldrich, Saint Louis, MO, USA). The calibration was prepared by processing the IAA solution in the same manner as the samples. The total IRC content, including IAA, was calculated using the equation generated from the standard curve. Means and standard deviations were calculated using three biological replicates. The measurements were taken every 24 h for 7 days of culture growth.

### 2.5. Estimation of the Yield and Content of Zinc, Iron, and Copper in the Grains 

The wheat grain yield (GY) was determined as follows: number of heads from 1 m^2^ × kernels/grain per head × kernels/grain weight. The following yield structure indicators were evaluated: thousand-grain weight (TGW), number of grains per spike (GPS), and the spike height (SH).

The concentrations of Zn, Fe, and Cu in the grains were determined using the atomic absorption spectroscopy method (AAS C115M1, Sumy, Ukraine) after microwave digestion in nitric acid (Sphera Sim, Lviv, Ukraine). A blank was included in each digestion batch for quality assurance. Mineral concentrations were expressed on a dry-weight basis. The microwave digestion of the plant material for the Zn, Fe, and Cu analysis was carried out using a microwave (Multiwave Go, Anton Paar, Graz, Austria) with a 12-vessel rotor 12HVT50. Approximately 0.9–1 g DW grains (oven-dried at 80 °C for 4 h) of each genotype were ground and transferred to the 50 mL 12HVT50 reaction pressure vessels. Subsequently, 6 mL of concentrated HNO_3_ and 3 mL of deionized H_2_O were added to complete a final volume of 9 mL. Following digestion, the digests were transferred into a 15 mL universal tube made up to a final volume of 15 mL by adding deionized H_2_O and stored at room temperature.

### 2.6. Statistical Analysis

The relationships between the studied factors were determined using regression analysis based on appropriate models and correlation coefficients. The significant influence of the factors used was assessed via the ANOVA significance test with repeated measures or an alternative nonparametric test. Differences with *p* < 0.05 were considered statistically significant. All statistical analyses, except the PCA analysis, were performed using Statistica 10 software.

Loading plots of elements and score plots of genotypes were drawn using the multivariate system of PCA in Past 4.03 software (https://past.en.lo4d.com/windows, accessed on 19 February 2021).

## 3. Results

### 3.1. Identification of Isolated Endophytes

Thirty-four microbial isolates were obtained from the studied wheat grains through the application of the culture-dependent protocol application. All isolates were assigned into groups with similar morphological features (Table 2).

Next, representative isolates were selected from these groups for further identification. Accordingly, we obtained 12 different isolates originating from the four varieties of spring wheat grains, which were assumed to belong to different taxonomic groups.

Eleven uniform isolates were obtained from the grains of the Oksamyt myronivs’kyi and Struna myronivs’ka varieties. Eight separate isolates were obtained from the Dubravka grains. The smallest number of uniform isolates (4) originated from the Holikovs’ka grains. From each group of isolates, three isolates representing a specific morphological group were selected for detailed identification. 

**Figure A1:**
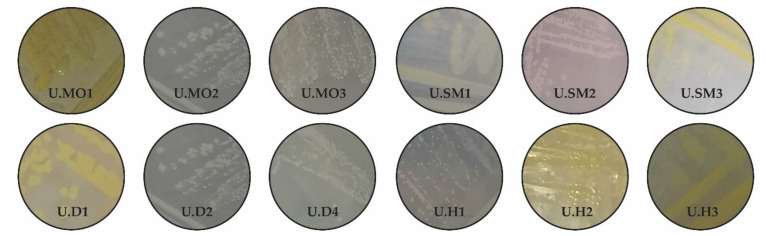
Identified isolates that were obtained from four varieties of spring wheat grains.

The isolated microorganisms displayed six types of pigmentation. Microorganisms isolated from the Oksamyt myronivs’kyi grains represented four types of pigmentation: yellow (36.5%), white (27.5%), beige (18%), and yellow-beige (18%). In Struna myronivs’ka, almost half of the samples had yellow (45.5%), yellow-cream (27.3%), beige (12.5%), or beige-pink (27.3%) pigmentation. Yellow-cream (25%), yellow-beige (37.5%), yellow (25%), and beige (12.5%) colonies were observed for microorganisms originating from the Dubravka grains. Three types of pigmentation were noted for the Holikovs’ka variety: yellow-cream (50%), beige (25%), and yellow (25%). Analysis within each variety revealed that the yellow pigmentation was the most abundant (Figure A1).

Using BLAST (Basic Local Alignment Search Tool) and the NCBI (National Center for Biotechnology Information) database, all isolates were identified at the genera level based on the 16S rRNA gene. It was found that the studied strains belonged to six genera (Table 3), namely, *Staphylococcus* (4), *Pantoea* (2), *Sphingobium* (2), *Bacillus* (2), *Kosakonia* (1), and *Micrococcus* (1).

The identification procedure revealed the presence of two strains from the genus *Pantoea* and one from *Staphylococcus* spp. in the variety Oksamyt myronivs’kyi. The identified strains that were obtained from the Struna myronivs’ka grains belonged to three different genera: *Kosakonia*, *Micrococcus*, and *Staphylococcus.* Out of four uniform isolates obtained from the Dubravka grains, we identified three strains belonging to the genera *Bacillus*, *Staphylococcus*, *and Sphingobium*. Using the culture-dependent protocol, we identified the same genera strains marked as U.H1, U.H2, and U.H3 in the variety Holikovs’ka (Table 3).

The molecular identification of the isolated endophytes with the use of NCBI BLAST revealed high sequence similarity of the identified bacteria to the genus *Pantoea* (GenBank: MT302200, MT302201) in the community isolated from *Amelanchier spicata* [78] (99.80%), *Sphingobium* (GenBank: MT302196, MT302198) in the population isolated from soil [79] (97.87%), *Kosakonia* (GenBank: MT302202) in the community isolated from rice rhizoplane [80] (98.80%), *Staphylococcus* (GenBank: MT302195, MT302197, MT302199, MT302204) in the population isolated from rice grain [81] (97.87%), *Micrococcus* (MT302203) in the community isolated from *Jatropha curcas* L. [82] (97.38%), and *Bacillus* (GenBank: MT302194, MT312840) isolated from *Alcyonium digitatum* [83] (97.51%).

### 3.2. Synthesis of the IRCs

**Figure 1:**
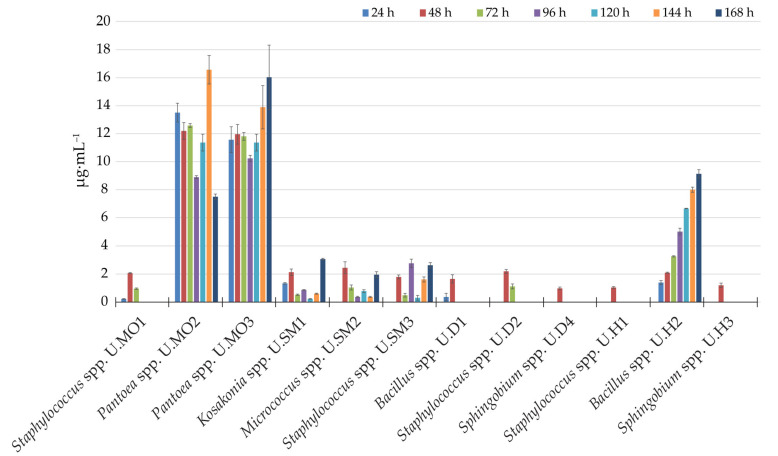
Concentration of indole-related compounds (IRCs, µg∙mL^−1^) that were detected using the Salkowski reagent in a liquid bacterial culture medium supplemented with 5 mM L-tryptophan. Samples were collected every 24 h.

All isolated bacterial strains demonstrated the ability to synthesize IRCs during in vitro growth in the presence of L-tryptophan, which is generally considered an IAA precursor (probably via the tryptophan-dependent pathway) [84]. For the qualitative assessment of IRCs, all isolates displaying the color change from light pink to pink upon the addition of the Salkowski reagent were considered positive for IAA. The amount of IRCs (µg∙mL^−1^) produced by the studied strains in the culture medium supplemented with 5 mM L-tryptophan is demonstrated in Figure 1.

The production of IRCs was estimated via differentiation in terms of the tested strain and its taxonomic features, as well as the duration of the experiment (*p* < 0.00001). In general, the studied isolates were characterized by significant differences in the ability to produce IRCs in the presence of L-tryptophan in a 168 h shaking culture (*p* < 0.00001). We demonstrated that strains belonging to the same genera, such as *Bacillus*, produced different amounts of IRCs. For example, *Bacillus* spp. U.D1 produced a low amount of IRCs after 24 and 48 h (0.36 and 1.65 µg∙mL^−1^), whereas *Bacillus* spp. UH2 produced significant levels of IRCs throughout the experiment (1.39–9.13 µg∙mL^−1^). We also found that the *Pantoea* spp. U.MO2, *Pantoea* spp. U.MO3, *Kosakonia* spp. U.SM1, *Micrococcus* spp. U.SM2, *Staphylococcus* spp. U.SM3, and *Bacillus* spp. UH2 strains continuously secreted IRCs in their culture medium (Figure 1). In contrast, several tested strains secreted IRCs only after 48 h of growth with L-tryptophan (strains: *Sphingobium* spp. U.D4—0.98 µg∙mL^−1^, *Staphylococcus* spp. U.H1—1.04 µg∙mL^−1^, and *Sphingobium* spp. U.H3—1.19 µg∙mL^−1^).

*Pantoea* spp. U.MO2 and U.MO3 secreted high amounts of IRCs at all time points of the experiment (Figure 1). The maximum IRC production for *Pantoea* spp. U.MO2 was found at 144 h with 16.57 µg∙mL^−1^ and the minimum was found at 168 h with 7.49 µg∙mL^−1^. In contrast to the U.MO2 strain, the U.MO3 strain enhanced its production of IRCs over time up to 168 h. The maximum production by this strain at a level of 16.04 µg∙mL^−1^ was observed after 168 h of the experiment. The lowest production of IRCs was observed in the following strains: *Staphylococcus* spp. U.MO1—0.22-2.07 µg∙mL^−1^, *Bacillus* spp. U.D1—0.36-1.65 µg∙mL^−1^, *Staphylococcus* spp. U.D2—1.12-2.19 µg∙mL^−1^, *Sphingobium* spp. U.D4—0.98 µg∙mL^−1^, *Staphylococcus* spp. U.H1—1.04 µg∙mL^−1^, and *Sphingobium* spp. U.H3—1.19 µg∙mL^−1^. The maximum production of IRCs was detected for *Pantoea* spp. U.MO2 with 16.57 µg∙mL^−1^ after 144 h. 

### 3.3. Grain Yields, Structure of the Harvest, and Concentrations of the Microelements

**Figure 2:**
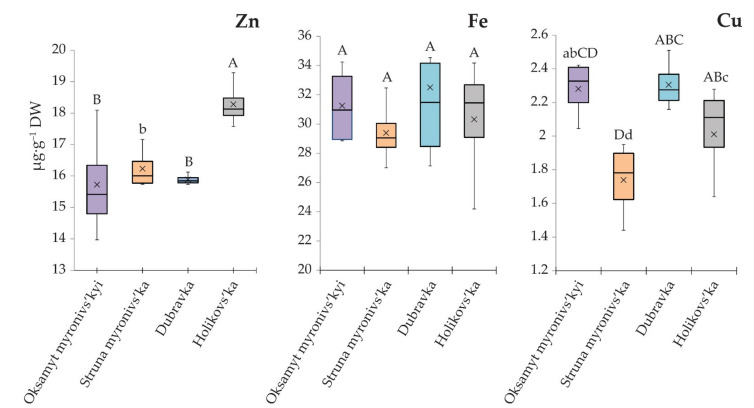
Grain Zn, Fe, and Cu concentrations (µg∙g^−1^ DW) at the stage of grain ripening. Wheat varieties were grown in the field with soil type Chernozem on eluvium of carbonate rock, pH 7.15, Dmytriv location (50°13′26.6″ N, 24°36′50.5″ E), in the Y2017 crop season. Statistically significant differences in the accumulation of individual elements were identified using Tukey’s test of one-way ANOVA. Levels not connected by the same letter are significantly different (capital letters indicate *p* < 0.05; lower case letters indicate *p* < 0.1).

Grain yield and grain nutritional quality depend on the interactions of numerous genes and environmental factors [85]. We examined the possible relationships between the concentration of micronutrients (Zn, Fe, Cu) in the grains and some yield characteristics, in particular, the number of grains per spike (GPS), spike height (SH), thousand-grain weight (TGW), and grain yield (GY) (Table 4, Figure 2).

Due to the varietal diversity and environmental differences, the relationships between the yield and its components are very complex [86]. The grain number per spike (GPS) has a significant effect on the thousand-grain weight (TGW) [87]. In our study GPS, TGW, and GY non-significantly differed between the three *T. aestivum* genotypes: Oksamyt myronivs’kyi, Struna myronivs’ka, and Dubravka (Table 4). Emmer Holikovs’ka was characterized by significantly lower yield attributes (27–34% for SH, 9–18% for GPS) in comparison with the bread varieties. The highest GY was recorded for the bread variety Oksamyt myronivs’kyi (63.26 qt∙ha^−1^), with a TGW of 40.01 g, followed by the Dubravka (60.66 qt∙ha^−1^) and Struna myronivs’ka (59.14 qt∙ha^−1^) varieties, and the lowest value was recorded for the Holikovs’ka variety (34.64 qt∙ha^−1^).

The concentration of micronutrients in plant tissues, and in grains in particular, depends on many factors, including the mineral concentration and bioavailability in the soil, soil pH, environmental conditions, agronomic management practices, and the ability of plants to transport these elements into harvested parts. The concentrations of Zn, Fe, and Cu in the grains of the studied wheat genotypes are presented in Figure 2.

There were evident significant differences in the micronutrient concentrations, except Fe, between the studied wheat varieties. Emmer Holikovs’ka was characterized by the highest grain concentration of Zn (18.28 µg∙g^−1^ DW), and average levels of Fe and Cu—30.32 µg∙g^−1^ DW and 2.01 µg∙g^−1^ DW, respectively. The genotype with the highest average Fe bioconcentration value in the grains was the variety Dubravka—32.49 µg∙g^−1^ DW, followed by var. Oksamyt myronivs’kyi—31.25 µg∙g^−1^ DW and var. Holikovs’ka—30.32 µg∙g^−1^ DW, while the lowest value was found for Struna myronivs’ka—29.39 µg∙g^−1^ DW. The maximum Cu concentration was noted for Oksamyt myronivs’kyi—2.40 µg∙g^−1^ DW and Dubravka—2.31 µg∙g^−1^ DW, while the minimum value was exhibited by Struna myronivs’ka—1.74 µg∙g^−1^ DW. Thus, there were significant differences between the concentrations of Zn and Cu in the grains of the studied genotypes caused by the low soil bioavailability of these elements and the efficacy of their acquisition by the studied varieties.

**Figure 3:**
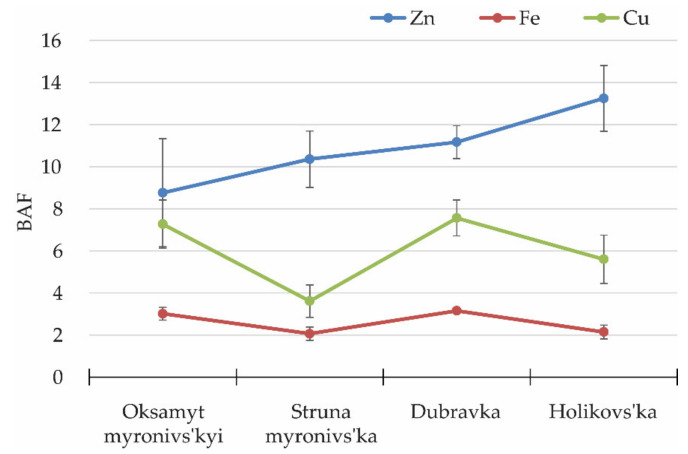
Biological accumulation factors (BAFs) of Zn, Fe, and Cu for bread and emmer spring wheat in field conditions on Chernozem soils on eluvium of carbonate rock, pH 7.15. Dmytriv location (50°13′26.6″ N, 24°36′50.5″ E), 2017 crop season.

Based on the soil minerals and their concentrations in the grains, we calculated the bioaccumulation coefficient or biological accumulation factor (BAF) for Zn, Fe, and Cu as the ratio of the concentration of the element in the grain to its bioavailable concentration in the soil. This index allows comparing the varietal ability to uptake and transport micronutrients to the grains (Figure 3). High Fe and Cu BAF values were noted for var. Dubravka and Oksamyt myronivs’kyi, whereas the maximum Zn BAF was obtained for var. Holikovs’ka.

A PCA biplot was constructed for the studied wheat genotypes; the analysis included the measures of TGW, GPS, and GY and concentrations of Zn, Fe, and Cu. The first two components, which explained the maximum cumulative variances of 0.94828%, were important (Table 5).

Among all the PCs, the first PC (0.62579%) contributed the most to the total variance. The major traits contributing to the first PC were Zn, GY, TGW, and GPS. In turn, Fe and Cu were the major contributors to the second PC. GPS and Cu were the diversity contributor traits in the third PC. 

**Figure 4:**
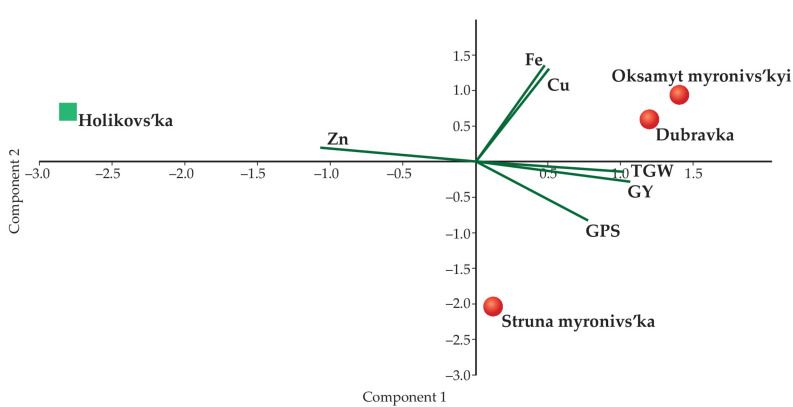
Principal component analysis (PCA) for the three micronutrients, grain yield (GY), number of grains per spike (GPS), and thousand-grain weight (TGW) in spring wheat (means for 2017).

The biplot explains the relationship of the four wheat genotypes with component traits (Figure 4). Across the genotypes, GY was positively associated with GPS, TGW, Cu, and Fe, and negatively associated with Zn. Fe and Cu were grouped together, and TGW, GY, and GPS were clustered together. The Dubravka and Oksamyt myronivs’kyi varieties were clustered together, whereas the Holikovs’ka variety was positioned distantly. The PCA biplot shows that emmer Holikovs’ka had a positive association with Zn. This means that the grain yield for Holikovs’ka was lower, but had a high micronutrient density, and Dubravka and Oksamyt myronivs’kyi were rich in Cu and Fe, in contrast to the Struna myronivs’ka grains.

## 4. Discussion

Our field studies were carried out in soil with low bioavailability of Zn, Cu, and Fe using wheat genotypes with different abilities to accumulate these micronutrients in grains. Low microelement bioavailability in soils is caused by high pH and high content of organic matter, the mineral and clay composition, porosity, and moisture content [60,88]. In plants, Zn is involved in carbohydrate metabolism [18] and auxin metabolism [19], acts as a potent antioxidant, and plays an important role in the normal development of floral tissues, flowering, fertilization, fruiting, and grain development [89,90]. Zn deficiency affects grain yield, pollen formation, root and leaf development, and water uptake and transport [19,91]. Most of the Zn-regulated enzymes are involved in the regulation of DNA transcription, RNA processing, and translation [89]. Fe is of great importance as well; as a redox-active metal, it is a component of many vital enzymes [92] and is thus involved in photosynthesis, mitochondrial respiration, nitrogen assimilation, biosynthesis of hormones (ethylene, gibberellic acid, jasmonic acid), DNA, production and scavenging of reactive oxygen species, osmoprotection, and pathogen defense [88,89]. A deficiency in bioavailable Fe in soil leads to a decline in photosynthesis [93], mitochondrial respiration, and protein structure formation [94], and results in poor plant growth and development [95,96]. Redox-active Cu is a cofactor for more enzymes that are involved in electron transfer reactions [97]. It is therefore involved in cell wall synthesis, photosynthesis, respiration, nitrogen metabolism, and oxidative stress protection [89]. Cu deficiency compromises plant fertility, stunts the growth of the whole plant, and limits plant productivity and grain production [17,20,97,98].

There are a great number of published experimental data on micronutrient (Cu, Fe, Mn, and Zn) concentrations in the grains of *T. aestivum* cultivars [2,3,4,5]. Different studies show a wide variation in grain Fe and Zn concentrations among wheat genotypes. The variations for Zn (32–57 ppm) and Fe (39–58 ppm) were observed among spring wheat genotypes by Chatrath et al. [99]. The Cu, Fe, and Zn concentrations in the grains of selected *T. aestivum* varieties were low, but within the ranges reported in the literature. We did not observe a positive correlation between the Fe and Zn concentrations, as described in numerous studies performed on bread wheat [100]. Published reports have indicated that wild and primitive wheats, such as *T. monococcum*, *T. dicoccon*, and *T. dicoccoides*, accumulate more Fe and Zn in grains than cultivated wheat and advanced lines [6,7,8]. Suchowilska et al. [16] reported concentrations of Fe, Zn, and Cu close to 49, 54, and 4.1 mg∙kg^−1^, respectively, and highlighted significant positive correlations between Fe, Zn, and Mn levels in *T. dicoccum* grains. Similarly, the mean concentrations of Fe, Zn, and Cu in emmer wheat lines were 41.72 mg∙kg^−1^, 17.06 mg∙kg^−1^, and 2.85 mg∙kg^−1^, respectively [15]. The concentrations of the microelements and TKW in the emmer wheat observed in this study agreed with the data reported in the literature. As shown by Zhao et al. [101], the mean concentrations in emmer wheat grain were as follows: Fe—34.1 mg∙kg^−1^ and Zn—22.8 mg∙kg^−1^. The mean TKW for the spring emmer accessions ranged from 22.9 to 42.6 g [102]. In this regard, it is important to emphasize that the concentrations of minerals in wheat grains depend on the uptake of microelements from the soil, their transport to the flag leaf, and further loading into the grain [103,104,105]. Such transport involves different members of the zinc-regulated transporters (ZRTs), iron-regulated transporter (IRT)-like protein (ZIP) family, heavy metal ATPases (HMAs) proteins of the P_1B_-type ATPase family, and members of the cation diffusion facilitator (CDF) family or yellow stripe-like (YSL) transporters [17,106,107]. 

Improvement of the wheat grain micronutrient quality via inoculation of endophytes from different genera appeared recently as one of the approaches for wheat biofortification [37,53,54,55,56,57,58,60]. Endophytic bacteria establish symbiotic, mutualistic, commensalistic, or trophobiotic interactions with various organisms, including plants [38]. In a field experiment, foliar application of endophytic bacteria increased the wheat height, leaf area, spike length, and plant biomass [108]. Consortia of endophytic microbes that exhibit mutual trophic relationships to each other also resulted in yield enhancement through an ≈30% increase in the number of spikelets, grains per spike, and grain yield per plant [109].

In our experiments, we observed statistically significant differences between the grain concentrations of Cu and Zn and the composition of isolated and bacterial endophytes identified in four spring wheat genotypes. The microorganisms represented the genera *Staphylococcus*, *Pantoea*, *Kosakonia*, *Micrococcus*, *Bacillus*, and *Sphingobium.* Our study confirmed that the microbiome of wheat grains depends on the wheat variety; the same trend was reported by Safin et al. [66] and Comby et al. [67]. Furthermore, Kuzniar et al. [68] found that the seed-borne microbiome was not statistically significantly dependent on the wheat cultivars. Importantly, the seed-associated microbiotas, which are expected to be transferred vertically, have the potential to coadapt with their host over generations to different harsh environments [69]. It was demonstrated [69] that the seed-associated bacteria of the domesticated bread wheat species *T. aestivum* were less diverse and more inconsistent among individual plants compared to those of the *T. dicoccoides* emmer wheat species in the wild. In contrast, we did not observe greater diversity among the isolates from the emmer Holikovs’ka grains compared to the isolates from the studied bread wheat varieties. The majority of the detected bacterial taxa had plant-growth-promoting effects on crops. Some bacteria from the genera detected in our study were reported as inoculants for Zn, Cu, or Fe biofortification of wheat [37,53,74].

All isolated bacterial strains synthesized different quantities of IRCs during in vitro growth. Woźniak et al. [110] suggested that almost all isolates are able to produce IAA, with concentrations that are dependent on the bacterial strain, genus, host plant, and the presence of an amino acid precursor. Moreover, Patern [111], Zahir et al. [112], and Passari et al. [113] frequently identified variations in the ability of plant-growth-promoting bacteria (PGPB) to produce IAA. These variations may be related to the locations of the genes involved, regulatory sequences, and the availability of enzymes that can modify active free IAA. Hardoim [114] reported that the key gene involved in the production of indole compounds is gene *ipdC*, encoding indole-3-pyruvate decarboxylase (EC, 4.1.1.74). The IPyA pathway has been detected in 34 genomes of endophytes, of which, 18 are from gammaproteobacterial strains; all *Kosakonia* strains in this study had a single copy of the *ipdC* gene [114]. Interestingly, Gross and Loper [115] reported that the *ipdC* gene is not detected in the genomes of *Pseudomonas*, i.e., common producers of auxins. 

IAA is recognized as the main effector molecule in phytostimulation, immunity, and the interaction between plants and bacteria [116]. Auxins produced by endophytic microbiota have an impact on micronutrient acquisition and transport processes by promoting rhizosphere acidification via the stimulation of H^+^-ATPase activity and controlling the expression of numerous genes that are important for nutrient homeostasis [58,116]. There is experimental evidence suggesting that auxin is involved in Fe and Zn homeostasis in graminaceous plants, but its specific role remains unclear. For instance, the rice transcription factor *Os*ARF12 activates the auxin response gene and affects Fe accumulation and distribution in rice [116]. In turn, *Os*ABCB1 is involved in auxin transport and Fe homeostasis [117]. Auxin regulates Fe-deficiency root responses that induce the release of siderophores in wheat [118] and influences wheat resistance to Fe toxicity [119]. Auxin signaling may also trigger Zn uptake and internal transport in rice under Zn deficiency [120]. Cu homeostasis is mainly regulated by transcription factors SPL7 and CITF1, which regulate Cu uptake into roots and delivery to flowers under Cu deficiency [121,122,123]. The involvement of auxins in these processes may be indirect by inducing changes in the JA level. It has been shown by Ishka and Vatamaniuk [98] that some symptoms of copper deficiency (increased shoot branching in Arabidopsis) can be rescued via the exogenous application of auxin. It is thought that an increase in plant growth also causes the increased release of root exudates for rhizobacterial metabolism. They can improve the grain yield of crops via promoting root growth [59,124,125]; photosynthesis; nutrient uptake, in particular Fe and Zn; their accumulation in plant tissues [25,37,61,125]. Unmasking the overall endophytic bacteria communities from wheat grains may help to identify and describe the microbial colonization of bread and emmer varieties, their link to the bioactive compounds produced [37,126], and their possible roles in mineral nutrition.

In our study, *Staphylococcus* spp. strains were isolated from the grains of each evaluated wheat genotype. Their influences on the plant microbiome and therefore on plants can differ from growth promotion to increasing tolerance to biotic and abiotic stresses. As reported by Jayakumar et al. [38], *Ceb1 Staphylococcus* sp. from the rhizome of *Curcuma longa* produce IAA and help to tolerate drought stress. *S. pasteuri* MBL_B3 from *Corchorus olitorius* exhibits growth promotion activity and is regarded as a prospective bioinoculant for jute plants [127]. Plant-growth-promoting *Staphylococcus* sp. bacteria from *Salicornia* sp. roots are able to produce ACC-deaminase and IAA. They reduce the harmful effects of salinity stress and their use as bioinoculants increases the wheat yield [128]. At the same time, some bacteria from this genus, for example, *S. aureus*, produce food toxins that cause human intoxication [129]. As indicated by our data, all isolated strains of genera *Staphylococcus* spp. were able to produce comparatively low quantities of auxins. Their specific roles in wheat metabolism need to be unraveled.

*Bacillus* spp. isolates were obtained from Dubravka and Holikovs’ka var. grains. Endophytes from the genus *Bacillus* are known to have a growth-promoting effect on wheat and are generally isolated from both grains and the rhizosphere [70,71,72,73]. As described in a review by White et al. [74], *Bacillus* sp. microbes possess high-affinity transporters that detect and absorb organic acid–metal complexes, acquiring mineral nutrients and carbon; in the plant root, cells extract metals from the microbes via the rhizophagy cycle. Singh et al. [37] inoculated wheat grains with endophytes *Bacillus subtilis* DS-178e and *Arthrobacter* sp. DS-179, which led to a twofold increase in Zn in grains, significantly promoting plant growth and grain yield in pot and field experiments [37,53]. There are also reports that many endophytic bacteria of the genus *Bacillus* show high activity against *Fusarium* species [130,131,132,133]. As reported by Pan et al. [72], *B. megaterium* (BM1) and *B. subtilis* (BS43, BSM0, BSM2) that were isolated from wheat grains had high antagonistic activity against *F. graminearum*. *B. gibsonii* and *B. pumilus*, which have antifungal properties, were isolated from *T. aestivum* [134]. Endophytes produce siderophores that bind to the available Fe, competing for this element with phytopathogens and protecting the host plant from diseases [40,135]. Zn solubilization by endophytes determines the plant’s intake amount in response to plant and microbial nutritional requests. Various plant-growth-promoting bacteria, including *B. aryabhattai* [61], *B. amyloliquefaciens*, *B. megaterium*, and *Bacillus* spp. [54,55], have shown Zn solubilization properties, as well as enhanced growth and zinc accumulation [56,57]. Concerning emmer wheat, there are available data on the isolation of fungal endophytes from *T. turgidum* ssp. *dicoccum* and *T. dicoccoides* [136,137,138]. To our knowledge, the current study is the first report on the isolation of endophytes from seeds of domesticated emmer wheat. We isolated two strains of the genus *Bacillus*, where one of them, namely, *Bacillus* spp. UH2, which was obtained from the Holikovs’ka variety, also produced a high quantity of IRCs during the experiment. Taking these data into account, we propose that the high Zn concentration in the emmer Holikovs’ka grain was in part related to the presence of *Bacillus* spp. endophytes. In turn, the presence of the *Bacillus* spp. U.D1 strain in the grain of the high-yielding Dubravka variety was not associated with Zn or bacterial IRC synthesis. 

In our experiment, bacteria from the *Pantoea* genus (Erwiniaceae family) were discovered only in the Oksamyt myronivs’kyi variety grains. This variety was characterized by a high grain yield and high Cu and Zn concentrations in the grains. The above characteristics, besides the varietal peculiarities, may result from the presence of *Pantoea* spp. microorganisms. These speculations are consistent with numerous published data on the growth-promoting, stress-tolerance-increasing, and zinc-solubilizing effects of *Pantoea* sp. endophytes that were isolated from wheat. As shown by Links et al. [139], these bacteria have antagonistic effects with seed-borne fungi. Chen et al. [50] reported that *Pantoea* sp. bacteria enhanced water stress tolerance in wheat. *P. agglomerans* strain Pa promotes seedling growth, increases chlorophyll content, lowers the accumulation of proline, and favours K^+^ accumulation in inoculated *Triticum durum* L. plants. It also produces secondary metabolites with salt stress alleviation and plant-growth-promoting activities. Therefore, this strain was proposed to be used as a biofertilizer for wheat in arid and salinity-affected regions [140]. *P. alhagi* has the ability to improve growth and drought tolerance in wheat [50]. Most reports have discussed *P. agglomerans* strains. *P. agglomerans* possesses many beneficial traits that can be used for the prevention and/or treatment of human and animal diseases and bioremediation of the environment [141]. To date, some of the yellow-pigmented, Gram-negative bacteria in the genus *Pantoea* have been used as commercial biocontrol products to control fire blight on apple and pear trees, such as BlightBan C9-1 and Bloomtime^TM^ Biological. Others have bioremediation potential, with the ability to degrade herbicides without generating toxic products. *P. dispersa* strains from sweet potato showed strong inhibition activity against the pathogenic fungus *Ceratocytis fimbriata* [142]. Interesting results were recorded for *P. dispersa* and *P. agglomerans* strains that were isolated from wheat [57]. These bacterial endophytes significantly increased shoot dry weight in pot experiments. *P. agglomerans* (EPS 17) produced a high level of IAA (8.449 µg∙mL^−1^) and their inoculation resulted in high Zn accumulation in the wheat roots, increasing the quantities of bioavailable Zn for plants and its mobilization toward wheat grains. In our experiments, the highest level of auxin production measured with the Salkowski reagent was recorded for *Pantoea* spp. U.MO2 MT302200 and *Pantoea* spp. U.MO3 MT302201. In this regard, the role of IAA on the activation of Fe-deficiency root responses in graminacea plants should be emphasized [112]. Furthermore, plants and microorganisms interact as a holobiome rather than as separate living organisms. IRC production in vitro might not reflect the in situ processes, which are dependent on other organisms from the whole endophytic community. We did not take into account the production of endogenous wheat auxins, which are also an important component of the wheat phytohormonal balance. Therefore, we can simply assume that indole-related compounds produced by *Pantoea* spp. or/and *Bacillus* spp. strains may be simply considered as one of the factors influencing the wheat yield formation and nutritional characteristics of the Oksamyt myronivs’kyi variety. 

A bacterial strain from the genus *Kosakonia* was isolated only from the Struna myronivs’ka var. grains. Plant-growth-promoting *Kosakonia radicincitan*s strains discovered recently in a variety of crops are known as factors that significantly influence grain yield and quality [143,144,145,146,147]. Other reports showed that *K. radicincitans* DSM 16656T (previously known as *Erwinia radicincitans* DSM 16656T and as *Pantoea agglomerans* D5/23) [143], which is associated with the phyllosphere of winter wheat, has the potential to biologically fix atmospheric nitrogen [144]. *P. agglomerans* was also reported as a *T. aestivum* root-growth-promoting agent [145]. The same strain was able to colonize both the rhizosphere and the phyllosphere of other cereal crops and to migrate within the plant. Increased root growth leads to improved water and minerals uptake, thereby increasing yields. As demonstrated by Becker et al. [146], the inoculation of winter wheat cv. Alcedo with *K. radicincitans* resulted in higher grain yields. *K. oryzae* EPS 7 isolated from wheat showed high levels of siderophore production and improved mineral nutrition [57]. Berger et al. [147] noted that *K. radicincitans* promoted the growth of radish plants. Nitrogen-fixing *Kosakonia* sp. ICB 117 from sugarcane and *K. radicincitans* DSM 16656T were able to produce auxins and cytokinins, promote plant growth, and increase the efficiency of plant metabolism [148,149]. Our findings confirmed the ability of *Kosakonia* spp. U.SM1 to secrete IRCs in vitro. However, in the case of the Struna myronivs’ka variety, we did not observe a high density of micronutrients in the grains, whereas the grain yield was comparatively high. Thus, we do not consider this strain as an instrument for biofortification purposes.

Another bacterial strain that was detected only in the Struna myronivs’ka grains was *Micrococcus* spp. U.SM2. This strain continuously secreted auxins (IRCs) into the culture medium for up to 168 h of cultivation in vitro. To date, we have found only two reports on isolating *Micrococcus* sp. endobacteria from *T. aestivum* grains [70,150]. As reported by Verma and coworkers [150], *M. luteus*, which is associated with wheat, has the ability to solubilize phosphorus and synthesize gibberellic acid. *M. luteus* bacteria establish a symbiosis with plants in the rhizophagy cycle [74]; these bacteria are part of the natural human skin flora and produce antimicrobial metabolites that exhibit probiotic properties [151]. There are reports concerning PGPB *Micrococcus* sp. from other plant sources, for example, TISTR2221, i.e., a cadmium-resistant strain from *Helianthus annuus* L., which produces a high level of IAA during the late stationary growth phase and increases the root length of maize seedlings under cadmium stress [152,153]. Raza and Faisal [154] found that *M. luteus*-chp37 inoculation increases the number of leaves, shoot length, root length, and weight of maize plants. *Micrococcus* sp. NII-0909 interacting with cowpea promotes plant growth and has the ability to produce IAA [155]. However, in the case of the Struna myronivs’ka variety, we did not observe a high density of micronutrients in the grains, whereas the grain yield was comparatively high. Thus, we do not consider these strains, i.e., U.SM1 and U.SM2, as an instrument for biofortification purposes. 

The endophytic bacteria *Sphingobium* spp. were isolated from wheat grains, e.g., U.D4 MT302196 from var. Dubravka and U.H3 MT302198 from var. Holikovs’ka, for the first time, and we confirmed that both strains produced small quantities of IRCs only for 48 h of the experiment. *Sphingomonas* sp. are common plant endophytes that are known to benefit plants by producing phytohormones and support plant maturation processes [156,157]. An increasing number of publications report the isolation of *Sphingobium* sp. from different sources: rice seeds [158] and the rhizosphere of peanut [159], *Ammophila breviligulata* [160], *Fortunella hindsii* [161], and maize [162]. They are found in roots, leaves, and flowers, and have been shown to play a protective role against phytopatogens [156,163] and water deficits (*Sphingomonas* sp. Cra20) [164]. Treatment of rice seeds with *S. yanoikuyae* MH394206 and *Azospirillum brasilense* enhanced the plant height, root volume, and the panicle and tiller quantity and increased the fresh weight of the rice seeds [165]. Molecular identification revealed the high sequence similarity of the cultured bacterial strain *Sphingobium* sp. (GenBank: MT302196, MT302198) to strain *Sphingobium* sp. SMB MK386690 that was isolated from soil [79]. Currently, there are available data on species belonging to Sphingomonadaceae (e.g., *Sphingomonas koreensis*) that are present in *T. durum* roots [166]. Xu et al. [167] demonstrated that the inoculation of wheat seeds with a strain of *Sphingomonas* spp. increased root biomass accumulation and the concentration of nutrients. Cadmium-immobilizing endophytic *Sphingomonas* sp. strain C40 from rice seeds decreased Cd availability and Cd grain uptake by increasing the pH and polyamine production in the host rice [158]. Hence, the N-fixing and the other above-mentioned traits of *Sphingomonas* spp. have become of particular interest, suggesting their possible role in plant growth promotion. Therefore, the *Sphingobium* spp. U.D4 MT302196 and U.H3 MT302198 roles in the plant–microbial–soil interaction, wheat yield, and mineral nutrition need to be studied. 

## 5. Conclusions

The presented results provide novel insights into the relationships between the grain endophytic bacteria, the Fe, Cu, and Zn concentrations, and the yield in the *T. aestivum* and *T. turgidum* subsp. *dicoccum* spring wheat varieties that were grown with limited bioavailability of these microelements in the field. The high-yielding Dubravka and Oksamyt myronivs’kyi bread varieties accumulated higher amounts of Fe, Cu, and Zn in grains when grown in a natural micronutrient-deficient environment on Chernozem carbonate soil with a high content of organic matter. The grain yield was positively associated with Cu and Fe and negatively correlated with the Zn concentration in grains across the studied genotypes. The emmer Holikovs’ka variety, with its lower yield capacity, was characterized by a high Zn bioaccumulation factor and a high concentration of this microelement in grains. We evidenced that the grains of the studied *T. aestivum* and *T. turgidum dicoccum* varieties were internally associated with a community of bacteria, some of which have the potential to be used as PGP inoculants for microelement biofortification purposes. The bacterial endophytes that were isolated from wheat grains belonged to the genera *Staphylococcus*, *Pantoea*, *Kosakonia*, *Micrococcus*, *Bacillus*, and *Sphingobium*, and their structure for each variety was different. For the first time, bacterial endophytes were isolated from grains of emmer *T. turgidum* subsp. *dicoccum* wheat. The indole-related compounds (auxins) that were produced by the endophytic bacterial genera *Pantoea* spp. U.MO2 and U.MO3 and *Bacillus* spp. U.H2 isolated from Oksamyt myronivs’kyi and Holikovs’ka grains may be regarded as one of the determinants of the wheat yield and its nutritional characteristics. *Pantoea* spp. U.MO2, U.MO3, and *Bacillus* spp. U.H2 isolates may have especially high potential as beneficial plant inoculants for nutrient-deficient agro-ecosystems. These microorganisms should be further tested for their ability to improve the yields of wheat and other crops and their nutritional quality.

## Figures and Tables

**Table 1:** Concentrations of DTPA-extractable micronutrients (mean and standard deviation values), pH, and content of organic matter in the soil (field experiment, 2017, Dmytriv village, Lviv region (50°13′26.6″ N, 24°36′50.5″ E)).

pH	Content of Organic Matter (g∙kg^−1^)	Micronutrients (ppm)		
		Zn	Fe	Cu
7.15 ± 0.02	74.59 ± 1.89	1.69 ± 0.21 (1.22–4.44)	12.46 ± 1.01 (8.23–18.29)	0.42 ± 0.05 (0.20–0.91)

**Table 2:** All bacterial isolates that were obtained from four varieties of spring wheat grains (abbreviation for identity: **+** yes, **-** no).

Isolation Source	All Isolates from the Studied Material	Colour of the Isolates	Number of Isolates Used for the Identification	Identity	Gram Staining
Oksamyt myronivs’kyi grain U.MO	11	Yellow Yellow	U.MO1	+	+
		White White White	U.MO2	+	-
		Beige Beige	U.MO3	+	-
		Yellow-beige Yellow-beige	U.MO4	-	
		Yellow Yellow	U.MO5	-	
Struna myronivs’ka grain U.SM	11	Yellow-cream Yellow-cream Yellow-cream	U.SM1	+	-
		Beige-pink	U.SM2	+	+
		Yellow Yellow Yellow Yellow Yellow	U.SM3	+	+
		Beige Beige	U.SM4	-	
Dubravka grain U.D	8	Yellow-cream Yellow-cream	U.D1	+	+
		Yellow-beige Yellow-beige Yellow-beige	U.D2	+	+
		Yellow Yellow	U.D3	-	
		Beige	U.D4	+	+
Holikovs’ka grain U.H	4	Beige	U.H1	+	+
		Yellow	U.H2	+	+
		Yellow-cream Yellow-cream	U.H3	+	-

**Table 3:** List of the identified bacterial endophytes (accession numbers from GenBank, NCBI) that were isolated from the grains of four varieties of spring wheat (field experiment, Dmytriv location (50°13′26.6′′ N 24°36′50.5′′ E), Y2017 crop season).

Isolation Source	Bacterial Genus	Culture Collection ID	GenBank Accession Number
Oksamyt myronivs’kyi grain	*Staphylococcus*	U.MO1	MT302199
	*Pantoea*	U.MO2	MT302200
	*Pantoea*	U.MO3	MT302201
Struna myronivs’ka grain	*Kosakonia*	U.SM1	MT302202
	*Micrococcus*	U.SM2	MT302203
	*Staphylococcus*	U.SM3	MT302204
Dubravka grain	*Bacillus*	U.D1	MT302194
	*Staphylococcus*	U.D2	MT302195
	*Sphingobium*	U.D4	MT302196
Holikovs’ka grain	*Staphylococcus*	U.H1	MT302197
	*Bacillus*	U.H2	MT312840
	*Sphingobium*	U.H3	MT302198

**Table 4:** Grain yield and yield attributes of spring wheat harvested at full maturity at the Dmytriv location (50°13′26.6″ N, 24°36′50.5″ E) in the 2017 crop season *. Tukey’s test of one-way ANOVA was used for analysis of statistically significant differences. Yield parameters indicated in each column were analysed separately. Levels not connected by the same letter are significantly different (capital letters indicate *p* < 0.05; lower case letters indicate *p* < 0.01; standard deviations and means were calculated using four replicates).

Wheat Variety	Number of Grains Per Spike (GPS)	Spike Height (SH) (mm)	Thousand-Grain Weight (TGW) (g)	Grain Yield (GY) (qt∙ha^−1^)
Oksamyt myronivs’kyi	32.52 ^AB^ ± 1.21	88.81 ^ABC^ ± 3.41	40.01 ^A^ ± 1.85	63.26 ^a^ ± 3.84
Struna myronivs’ka	35.77 ^A^ ± 1.04	85.84 ^AB^ ± 3.55	37.83 ^AB^ ± 0.92	59.14 ^a^ ± 3.21
Dubravka	35.62 ^A^ ± 1.93	78.93 ^AC^ ± 2.53	37.46 ^AB^ ± 1.05	60.66 ^a^ ± 2.12
Holikovs’ka	29.57 ^B^ ± 0.94	58.02 ^D^ ± 1.34	33.29 ^B^ ± 1.18	34.64 ^b^ ± 3.54

**Table 5:** Vector loadings and percentage variations that were explained by the three principal components (PC).

Parameters	PC1	PC2	PC3
GPS	0.72112	−0.55793	0.41075
TGW	0.93699	−0.092344	−0.33693
GY	0.97985	−0.19252	−0.053142
Fe	0.44635	0.89224	0.06846
Cu	0.46273	0.87517	0.14126
Zn	−0.99163	0.12679	0.024547
Loadings			
Eigenvalue	3.75474	1.93496	0.310304
Percentage variance	0.62579	0.32249	0.051717

## Data Availability

The additional data are contained within the article as Appendix A.

## Appendix A

**Table A1:** Quality and quantity of DNA used in the PCR reaction. Data are presented as means (*n* = 3).

Culture Collection ID	Mean Concentration of DNA (µg·mL^−1^) ± SD	Mean A 260:280 Ratio ± SD	Mean A 260:230 Ratio ± SD
U.MO1	1202.02 ± 2.95	2.07 ± 0.00	1.97 ± 0.00
U.MO2	1278.19 ± 6.61	1.99 ± 0.00	1.75 ± 0.00
U.MO3	1010.06 ± 1.86	2.05 ± 0.00	1.87 ± 0.00
U.SM1	914.39 ± 1.55	2.06 ± 0.00	1.67 ± 0.00
U.SM2	708.86 ± 0.20	1.93 ± 0.00	1.04 ± 0.00
U.SM3	272.88 ± 0.28	1.92 ± 0.00	1.35 ± 0.00
U.D1	508.06 ± 0.34	2.01 ± 0.00	1.94 ± 0.00
U.D2	542.37 ± 0.74	1.94 ± 0.01	0.94 ± 0.00
U.D4	389.67 ± 1.23	1.99 ± 0.00	1.31 ± 0.01
U.H1	96.75 ± 0.76	1.78 ± 0.00	1.08 ± 0.01
U.H2	277.72 ± 0.46	1.97 ± 0.00	1.62 ± 0.00
U.H3	357.57 ± 0.71	1.88 ± 0.00	1.64 ± 0.00

**Table A2:** Primers used in the studies.

Primer (5′–3′)		References
27F	AGAGTTTGATCATGGCTCAG	[168]
1942R	TACCTTGTTACGACTT	[169]
